# Quantitative and qualitative sex difference in habenula-induced inhibition of midbrain dopamine neurons in the rat

**DOI:** 10.3389/fnbeh.2023.1289407

**Published:** 2023-11-13

**Authors:** Dana Bell, Vaughn J. Waldron, P. Leon Brown

**Affiliations:** Maryland Psychiatric Research Center, Department of Psychiatry, University of Maryland School of Medicine, Baltimore, MD, United States

**Keywords:** LHb, RMTg, tVTA, prediction error, RPE, SABV

## Abstract

**Introduction:**

Clinically relevant sex differences have been noted in a number of affective, behavioral, cognitive, and neurological health disorders. Midbrain dopamine neurons are implicated in several of these same disorders and consequently are under investigation for their potential role in the manifestation of these sex differences. The lateral habenula exerts significant inhibitory control over dopamine neuronal firing, yet little is known about sex differences in this particular neurocircuit.

**Methods:**

We performed *in vivo*, single unit, extracellular recordings of dopamine neurons in female and male anesthetized rats in response to single pulse stimulation of the lateral habenula. In addition, we assessed baseline firing properties of lateral habenula neurons and, by immunochemical means, assessed the distribution of estrogen receptor alpha cells in the lateral habenula.

**Results:**

Habenula-induced inhibition of dopamine neuronal firing is reduced in female rats relative to male rats. In addition, male rats had a higher prevalence of rebound excitation. Furthermore, the firing pattern of lateral habenula neurons was less variable in female rats, and female rats had a higher density of estrogen receptor alpha positive cells in the lateral habenula.

**Discussion:**

We found that the dopamine neuronal response to habenular stimulation is both qualitatively and quantitatively different in female and male rats. These novel findings together with reports in the contemporary literature lead us to posit that the sex difference in dopamine inhibition seen here relate to differential firing properties of lateral habenula neurons resulting from the presence of sex hormones. Further work is needed to test this hypothesis, which may have implications for understanding the etiology of several mental health disorders including depression, schizophrenia, and addiction.

## 1. Introduction

Phasic changes in the firing rate of midbrain dopamine (DA) neurons follow the presentation of motivationally salient stimuli, leading to transient changes in neurotransmitter release that help optimize action selection (Schultz, 2010). Acute aversive events in general inhibit DA neuronal firing (Ungless et al., 2004), a neurophysiological outcome that can be replicated by activation of the lateral habenula (LHb) (Ji and Shepard, 2007). The LHb is one component of a neurocircuit that signals aversive events (Stamatakis and Stuber, 2012; Lawson et al., 2014), primarily through activation of the rostromedial tegmental nucleus (RMTg) (Jhou et al., 2009; Brown and Shepard, 2013), which in turn inhibits DA neurons (Brown et al., 2017). Transient cessations in DA neuronal firing are key to error prediction based theories of associative learning (Roesch et al., 2012) and impairments in this neurocircuit lead to impaired optimization of learned behavior (Shumake et al., 2010). Alterations in the fidelity of associative learning are relevant to understanding not only addiction (Keiflin and Janak, 2015) but also affective disorders (Whitton et al., 2015), the behavioral side-effects of Parkinson’s disease treatment (Lee and Jeon, 2014), and schizophrenia (Conn et al., 2020). As such, understanding the functionality of the LHb-RMTg-DA pathway has broad applications to the field of neurological and mental health.

Clinically relevant sex differences have been noted in the prevalence or severity of several of the conditions listed above. For example, men are more likely to initiate and abuse drugs (Becker, 2016) while women proceed more rapidly to addiction (Hernandez-Avila et al., 2004) and experience more craving in response to drug cues (Kennedy et al., 2013). Parkinson’s disease is more prevalent in men, but women have a faster progression and higher mortality rate (Cerri et al., 2019). Furthermore, there are well-documented higher diagnoses rates of depression in women (Rubinow and Schmidt, 2019) and schizophrenia in men (Goldstein et al., 2013). In each of these conditions, modulation of DA function by sex hormones may be a significant factor contributing to these differences (Becker, 2016; Litim et al., 2016; Laman-Maharg and Trainor, 2017; Searles et al., 2018).

The LHb, with its ability to regulate DA neuronal firing, has also been implicated in these same conditions (Hikosaka, 2010; Han et al., 2014; Stopper and Floresco, 2015; Hu et al., 2020). Despite this, relatively little work has been conducted on potential sex differences in the function of the LHb-RMTg-DA pathway. Early work in rats showed that estrogen-mediated activation of the LHb is key to the continued expression (Lonstein et al., 2000), though not the onset (Matthews Felton et al., 1999), of maternal behavior. More recent work in mice has demonstrated a role for the LHb in regulating sex differences in social communication (Rigney et al., 2020), parental behavior (Lecca et al., 2023) and in the susceptibility to sub-chronic variable stress (Zhang et al., 2018b). Melis et al. (2013) demonstrated that activation of the RMTg in male rats results in greater inhibition of DA neurons than in females. This study was limited, however, in that only nucleus accumbens-projecting ventral tegmental area (VTA) DA neurons were recorded and inhibition was only found in ~25–30% of neurons rather than the >80% generally seen with activation of the LHb (Christoph et al., 1986; Ji and Shepard, 2007; Brown et al., 2017). It is therefore unclear whether this outcome applies in general to the LHb-RMTg-DA pathway. To further elucidate potential sex differences, we used a combination of *in vivo* electrophysiology and immunochemistry in rats to test the hypothesis that the inhibitory strength of this neurocircuit is more pronounced in males than females.

## 2. Materials and methods

### 2.1. Animals

Adult Sprague–Dawley rats (225–300 g upon arrival; Charles River Laboratories; Wilmington, MA) were delivered to the animal facilities at the Maryland Psychiatric Research Center and maintained on a 12:12 h light:dark cycle (lights on at 0600) with food and water *ad libitum*. All animals were given a minimum 48-h period of acclimation prior to experiments. For electrophysiology, 34 rats (17 female and 17 male) were used for DA extracellular recording with a mean of 3.9 neurons recorded from each animal, and 30 rats (15 female and 15 male) were used for LHb extracellular recordings with a mean of 3.4 neurons recorded from each animal. Immunochemistry was performed on well-preserved tissue from rats used in the electrophysiology experiments. Thirteen rats (8 female and 5 male) were used for RMTg neuronal counts while 19 rats (11 female and 8 male) were used for LHb estrogen receptor alpha (ERα) cell counts. This study was conducted in strict accordance with recommendations from The Guide for the Care and Use of Laboratory Animals (National Research Council, 2011). All procedures were approved by the University of Maryland School of Medicine Institutional Animal Care and Use Committee.

### 2.2. *In vivo* electrophysiology

Rats were anesthetized with urethane (1.5 g/kg, ip; Sigma-Aldrich; St. Louis, MO) with additional ip injections of urethane delivered as needed to maintain the plane of anesthesia. Tissues surrounding the ear canals and wound margins were infiltrated with 2% mepivacaine, and the rat was placed in a stereotaxic instrument with atraumatic earbars (David Kopf Instruments; Tujunga, CA). Body temperature was maintained at 37°C using a feedback-controlled heating pad.

#### 2.2.1. DA neuronal recordings

The scalp was incised, and a rectangular skull section was removed to expose the dorsal brain surface of the right hemisphere above the LHb and midbrain (RC: −3.0 to −6.5 mm; ML: 0.0 to 3.0 mm; all coordinates obtained from Paxinos and Watson (2007)). The dura was carefully removed for the insertion of stimulating and recording electrodes. A concentric, bipolar stimulating electrode (SNEX-100X; Microprobes; Gaithersburg, MD) was lowered into the LHb (RC: −3.5 mm; ML: 1.5 mm; DV: 5.2 mm @ 10°). Recording electrodes were prepared from borosilicate glass capillary tubing (1.5 mm outer diameter, BF 1401; World Precision Instruments; Sarasota, FL) using a vertical puller (PE-2; Narishige; Amityville, NY) and filled with 2 M NaCl. Tips were broken back to create microelectrodes with an *in vitro* impedance of ~5–8 MΩ. Recording electrodes were attached to either a piezoelectric (Inchworm; Burleigh; Fishers, NY) or hydraulic (Trent Wells; Coulterville, CA) microdrive and lowered through the midbrain substantia nigra (SN) and VTA (Range RC: −5.4 mm to −6.2 mm; ML: 1.0 to 2.5 mm; DV: −6.8 to −8.5 mm) until a spontaneously active cell was isolated from background noise. Electrode potentials were amplified, filtered (0.1–8 kHz bandpass), and monitored in real time using a digital oscilloscope and audiomonitor. Electrical activity was digitized at 20 kHz using a laboratory interface (micro1401; CED; Cambridge, England) and stored on disk for offline analysis using Spike 2 software (CED; Cambridge, England). Putative DA neurons were identified electrophysiologically (Ungless and Grace, 2012; Brown et al., 2017) and once isolated, a baseline of approximately 500 spike events was recorded. Following this, neurons were tested for their response to repeated application of rectangular current pulses (biphasic, 1.0 mA, 100 μsec, 0.5 Hz) to the LHb. A minimum of 500 spikes was collected during LHb stimulation. Up to six recording tracks were made during the course of an experiment, each separated by a distance of 500 μm. At the end of the recording session, a glass recording electrode filled with 1 M NaCl and saturated with fast green was lowered to the coordinate of the last recorded neuron, and fast green was iontophoretically ejected from the pipette tip (−25 μA, 60 min) to later calculate the position of previously recorded neurons. The position of the stimulating electrode was marked by passing a constant DC current (−0.1 mA, 8 s).

#### 2.2.2. LHb neuronal recordings

Extracellular recordings from the LHb followed our previous protocol (Wang et al., 2015), which was similar to the DA recordings with the following exceptions. No stimulating electrodes were positioned in the LHb of recorded animals. After incising the scalp, a burr hole was made overlying the LHb (RC: −3.5 mm; ML: 0.9–1.5 mm). Recording electrodes filled with 1 M NaCl and saturated with fast green (impedance ~8–15 MΩ) were slowly advanced along a track through the dorsal-ventral extent of the LHb at a 10° angle. A total of three tracks were made separated laterally by 300 μm. Isolated spiking cells were recorded for a minimum of 500 spike events before continuing along the track. At the end of experiment, the final position of the recording electrode was marked by iontophoretically ejected fast green (−25 μA, 60 min) to determine the position of previously recorded neurons.

### 2.3. Histology

Following electrophysiological recordings, rats were deeply anesthetized and perfused transcardially with 100 mL of 4° C phosphate buffered saline (PBS) followed by 500 mL of 6% formalin (pH 7.4, 4° C). Brains were rapidly removed and post-fixed overnight in preparation for histology. Brains were then equilibrated with a solution of 30% sucrose in PBS and sectioned in the coronal plane at 40 μm on a cryostat (CM 3050S; Leica; Deer Park, IL) through the rostral-caudal extent of the LHb and RMTg in five series with the result that each series contained sections 200 μm apart. Each series was placed in cryoprotectant liquid and stored at 4° C.

#### 2.3.1. Confirmation of electrode placement

One series of sections was slide mounted and counterstained with 0.1% neutral red for contrast to determine electrode locations. Photomicrographs of LHb and midbrain sections were captured digitally using either an Olympus BX41 or VS200 slide scanner (Olympus; Center Valley, PA) for offline analysis. Photomicrographs were overlaid on corresponding sections from a rat atlas (Paxinos and Watson, 2007) to reconstruct the location of each recorded cell and trajectory of each track. Only rats with stimulating probes (if used) that were placed within the boundaries of the LHb as determined from electrolytic lesions and with recorded neurons within the target area (SN/VTA or LHb) as determined by dye spot locations were used for electrophysiological analysis.

#### 2.3.2. RMTg NeuN immunochemistry

A second series of sections was used to determine neuronal cell counts in the RMTg by immunohistochemical localization of the neuron-specific protein NeuN. Sections were incubated at room temperature successively, with 3 PBS rinses following each step, in (1) 0.3% H_2_O_2_ in PBS for 30 min, (2) rabbit anti-NeuN polyclonal primary antibody ([1:10,000]; ABN 78, Millipore; Burlington, MA), 3.0% normal goat serum (NGS), 0.3% Triton-X in PBS overnight, (3) biotinylated goat anti-rabbit secondary antibody ([1:600]; BA-1000, Vector Laboratories; Newark, CA), 1.0% NGS, 0.3% Triton-X in PBS for 30 min, then directly into (4) avidin-biotin immunoperoxidase (Vectastain elite, PK-6100; Vector Laboratories; Newark, CA) in PBS for 30 min, and then directly into (5) 0.03% 3–3′-diaminobenzidine (DAB; Sigma-Aldrich; St. Louis, MO) in PBS for 2–5 min. Omission of the primary antibody in one section was used as a negative control in all incubations. All sections were mounted on glass slides, dried overnight, and coverslipped. Photomicrographs of brain sections were overlaid on templates delineating the boundaries of the bilateral RMTg through its rostral-caudal extent (Jhou et al., 2013; Brown et al., 2017); all positively stained, ovular objects within the bounds of the RMTg were counted.

#### 2.3.3. LHb ERα immunochemistry

A third series of sections was used to determine ERα + cell counts within the LHb. Sections were incubated at room temperature successively in (1) three rinses with PBS, (2) 0.3% H_2_O_2_ in PBS for 30 min, (3) three rinses with 0.3%Triton-X in PBS, (4) 3.0% NGS, 0.3% Triton-X in PBS for two hours, (5) rabbit anti-ERα polyclonal primary antibody [(1:10,000);06–935, Millipore; Burlington, MA], 3.0% NGS, 0.3% Triton-X in PBS overnight, (6) three rinses with 0.3%Triton-X in PBS, (7) biotinylated goat anti-rabbit secondary antibody [(1:600); BA-1000, Vector Laboratories; Newark, CA], 1.0% NGS, 0.3% Triton-X in PBS for 90 min, (8) three rinses with PBS, (9) avidin-biotin immunoperoxidase (Vectastain elite, PK-6100; Vector Laboratories; Newark, CA) in PBS for 30 min, (10) three rinses with PBS, and (11) 0.03% 3–3′-diaminobenzidine (DAB; Sigma-Aldrich; St. Louis, MO), 0.02% nickel ammonium sulfate in PBS adjusted to pH 7.1 for 2–5 min. Omission of the primary antibody in one section was used as a negative control in all incubations. All sections were mounted on glass slides, dried overnight, and coverslipped. Photomicrographs of stained LHb sections were digitally captured and overlaid with unilateral templates derived from Paxinos and Watson (2007); all positively stained ovular objects within the bounds of the LHb were counted.

### 2.4. Electrophysiological analysis

Recorded sessions were analyzed and, using the waveform sorting algorithm provided in Spike 2, individual spikes were isolated from background noise and stimulus artifacts.

#### 2.4.1. DA neuronal analysis

The analysis of firing properties was similar to our previously used procedure (Brown et al., 2017). Basal firing properties were obtained from the initial baseline recording period and included firing rate, averaged waveform shape, and distribution of interspike interval (ISI), which was used to calculate the coefficient of variation (CV) of ISI, a measure of the regularity of neuronal firing (Chergui et al., 1993; Johnson, 1996; Iyer et al., 2017). An additional analysis of regularity, firing pattern, was performed using autocorrelograms with a 2 s time window and 5 ms bin widths, which were generated from spontaneous firing during baseline recording. Neurons were classified as burst firing if their autocorrelogram displayed a rapid rise with peak in the first 80 ms, followed by a trough and return to steady state. To determine bursting parameters, bouts of bursting activity (initial spike pair with an ISI ≤ 80 ms and terminal spike pair with an ISI > 160 ms) were calculated. Only neurons that exhibited a minimum of three, three-spike bursts over the course of 500 spikes were classified as bursting neurons. Neurons exhibiting three or more equally spaced peaks in the autocorrelogram occurring at integral multiples of the mean ISI were classified as pacemaker neurons. After separating out all burst-firing and pacemaker neurons, all remaining neurons, which had autocorrelograms with a rapid rise to a steady state, were classified as irregular neurons (Brown et al., 2017).

Peri-stimulus time histograms (PSTH) of spike events occurring 0.5 s before and 1.5 s after LHb stimulation were compiled using a 1 ms binwidth. To analyze the evoked response of individual neurons, cumulative summation plots were constructed from PSTHs by adding the contents of each bin to a running sum of all previous events. Neurons were classified as displaying excitation, inhibition, or no change for both the initial and secondary response to LHb stimulation as previously described (Ji and Shepard, 2007). To analyze overall evoked response, a mean PSTH was compiled from all neurons within a group as previously described (Brown et al., 2017) and smoothed by calculating a 25-point exponential weighted moving average (EWMA) using the formula:


$$EWMA_{t}=\left(\alpha *FR_{t}\right)+\left(1-\alpha \right)*EWMA_{t-1})$$

where *t* is any given time point, α is the weighting factor [2/(1 + 25)], FR_t_ is the firing rate at time point *t*, and given that EWMA_0_ is equal to a simple moving average of the first 25 ms of the histogram. Group differences (EWMA_Female_ − EWMA_Male_) were calculated at each time point as were the mean and standard deviation for group differences in the 0.5 s immediately before LHb stimulation.

#### 2.4.2. LHb neuronal analysis

Baseline firing activity was analyzed in a manner similar to that of DA neurons, resulting in measures of firing rate, firing pattern, ISI, and CV of ISI. Neurons were classified as burst firing if their autocorrelogram displayed a rapid rise with peak in the first 20 ms, followed by a trough and return to steady state. To determine bursting parameters, bouts of bursting activity (initial spike pair with an ISI ≤ 20 ms and terminal spike pair with an ISI > 100 ms) were calculated (Yang et al., 2018). Neurons were classified as tonic firing if their autocorrelogram displayed three or more equally spaced peaks or a rapid rise to a steady state (pacemaker or irregular, respectively).

### 2.5. Statistical analysis

Data were analyzed in a blind manner. Unless otherwise stated, all data are expressed as the arithmetic mean ± standard error of the mean. All statistical tests were performed using Sigmaplot. Categorical data were analyzed using chi-square tests. Ordinal data were analyzed with Mann–Whitney U tests. Distributions were compared with a Kolmogorov–Smirnov test. Multiple linear regression was performed to test for the effect of sex while accounting for recording location, with a Box-Cox transformation applied to non-normally distributed data. Pearson’s r was used to determine correlation coefficients. All other data were analyzed by t-test or ANOVA with post-hoc Tukey tests.

## 3. Results

### 3.1. Duration of LHb-induced inhibition of DA neurons differs by sex

**Figure 1:**
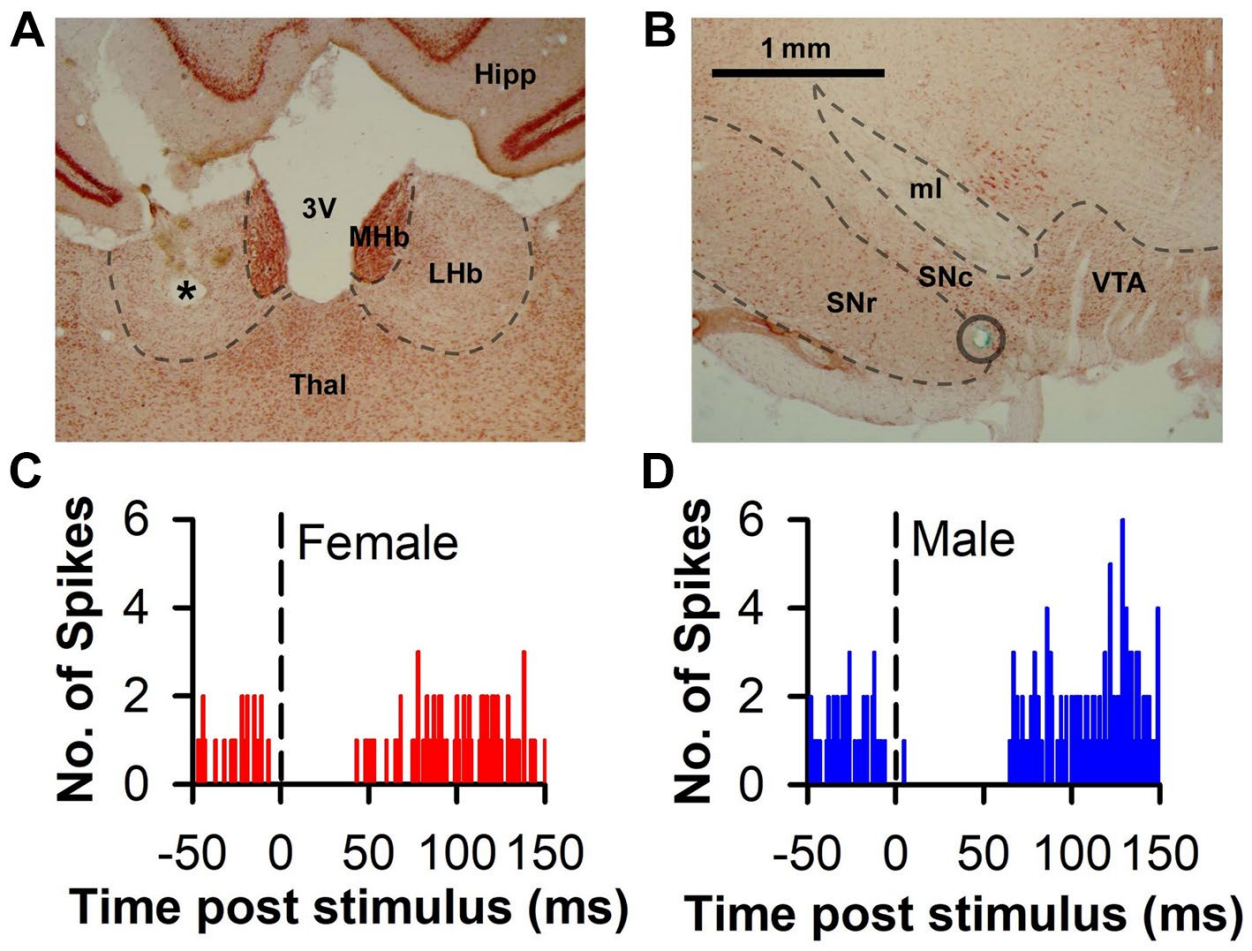
Representative histology and PSTH associated with LHb-induced DA inhibition. **(A)** Representative photomicrograph of an electrolytic lesion (*) from a stimulating electrode placed within the LHb and counterstained with neutral red. **(B)** Representative photomicrograph of DA area used for recording. The blue-green dye mark (circle) was iontophoretically released at the end of the recording track after having passed through the SN. **(C,D)** Representative histograms of raw spike counts of DA neurons inhibited by LHb stimulation in both a female and male rat, respectively. 3 V, third ventricle; Hipp, hippocampus; LHb, lateral habenula; MHb, medial habenula; ml, medial lemniscus; SNc, substantia nigra pars compacta; SNr, substantia nigra pars reticulata; Thal, thalamus; VTA, ventral tegmental area. Scale bar = 1 mm **(A,B)**.

DA recordings were obtained from a total of 133 neurons (71 from female and 62 from male rats) in the SN or lateral VTA; neurons were only retained for analysis from rats with the stimulating electrode histologically confirmed to be within the anatomical bounds of the LHb (Figures 1A,B). Basal firing rates did not differ between neurons from females and males [*t*_(131)_ = 0.84; *p* = 0.40; Table 1]. While the median CV of ISI was higher in neurons from males this difference was not significant (Mann–Whitney *U* = 519, *p* = 0.075). Similarly, though higher in neurons from males, the difference in prevalence of burst firing was not significant (*Χ*^2^ = 3.41, *p* = 0.065).

**Table 1:** Baseline firing properties and evoked response in DA neurons.

Variable	Female (*n* = 71)	Male (*n* = 62)
Firing rate, Hz ± SEM	4.5 ± 0.22	4.7 ± 0.25
Median CV of ISI, % (IQR)	35.0 (20.5–47.3)	45.8 (30.6–59.3)
Burst firing, %	34.2 (13/38)	55.6 (20/36)
Inhibitory response, %	83.1 (59/71)	88.7 (55/62)
Rebound excitation, %	**39.0 (23/59)**	**60.0 (33/55)**
Duration of inhibition, ms ± SEM	**47.0 ± 3.1**	**62.0 ± 3.7**

Data in bold represent a significant group difference (see text for details).

**Figure 2:**
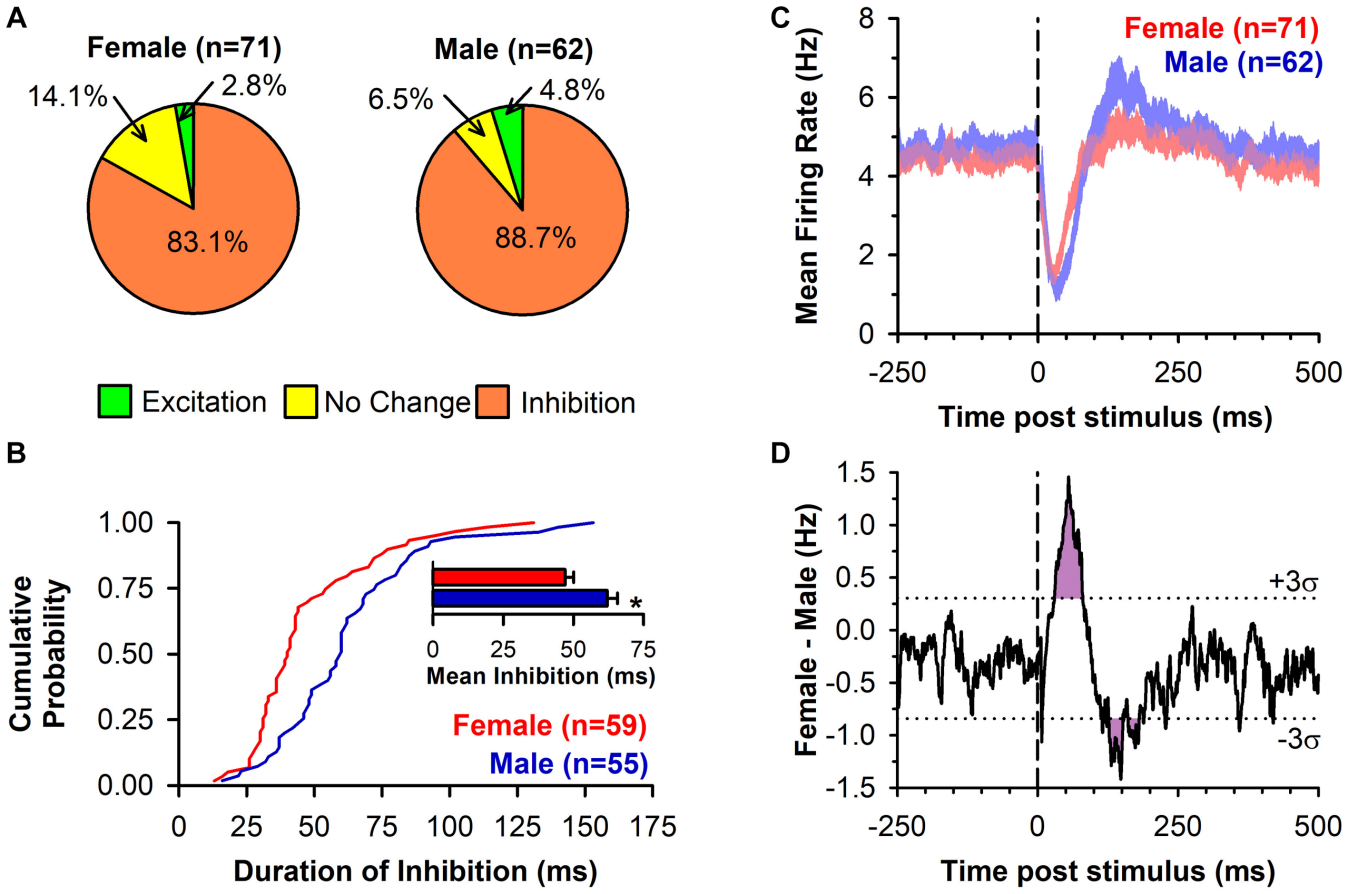
LHb-induced inhibition of DA neuronal firing is of longer duration in male rats. **(A)** Pie charts displaying the DA neuronal response to LHb stimulation by type in both female and male rats. **(B)** Cumulative probability graph showing the distribution of the duration of inhibition of DA neuron firing following LHb stimulation. Female rats (red line) had a distribution significantly shifted to the left of male rats (blue line; *p* < 0.001). Inset bar graph shows the mean duration of inhibition of DA neurons from female rats (red) and male rats (blue; *, *p* = 0.002). **(C)** PSTH (mean EWMA ± SEM, see methods for detail) for all recorded neurons relative to LHb stimulation (dashed line), regardless of response type, for both female rats (red) and male rats (blue). Overlap between groups is in purple. **(D)** Group difference from PSTH in C (black line) with ± three standard deviations (dotted lines) of the baseline difference score overlaid. Both the sex difference in inhibition and rebound excitation exceeded three standard deviations for an extended period (purple shading).

In response to single pulse LHb stimulation, the predominant response of DA neuron firing was inhibition (Figures 1C,D). This inhibition was prevalent in neurons from both females (83.1%) and males (88.7%) with the remainder of neurons displaying either no change or excitation (Figure 2A). There was no significant difference between sex in the prevalence of the neuronal inhibitory response (*Χ*^2^ = 0.85, *p* = 0.36). However, amongst neurons that were inhibited, the duration of inhibition was significantly longer in neurons from males relative to females, a difference that was apparent both as a significant separation in the distribution of duration of inhibition (Kolmogorov–Smirnov test, *d* = 0.44, *p* < 0.001; Figure 2B) and as a significant difference in mean duration of inhibition [*t*_(112)_ = 3.13, *p* = 0.002; Figure 2B inset]. This sex difference remained when averaging mean duration within subjects to account for clustering effects (Galbraith et al., 2010) with male rats having a significantly longer inhibition than female rats [*t*_(32)_ = 3.35, *p* = 0.003].

While the majority phenotype amongst DA neurons in response to LHb stimulation was inhibition of firing, this did not necessarily reflect the population effect, as there were neurons that showed other responses or that may have shown sub-threshold levels of inhibition. To capture the overall effect of LHb stimulation, a mean PSTH of all recorded neuronal responses was constructed (Figure 2C). This showed a clear inhibition following LHb stimulation in both sexes, but one that was more pronounced in males, along with a delayed rebound excitation that was more prominent in males. Indeed, within the category of inhibited neurons there was a significantly lower prevalence of rebound excitation in DA neurons from female compared to male rats (38.9% vs. 60.0% respectively; *Χ*^2^ = 5.03, *p* = 0.025). These sex differences can be more clearly seen by the difference in mean firing rates (Figure 2D), which exceeded three standard deviations (i.e., was further from the mean difference than 99.7% of the data) during the post-stimulation periods from 30 to 80 ms and from 127 to 180 ms (with the exception of 152–160 ms). This corresponded to the periods during which neurons from males had greater initial inhibition and a greater rebound excitation, respectively.

**Figure 3:**
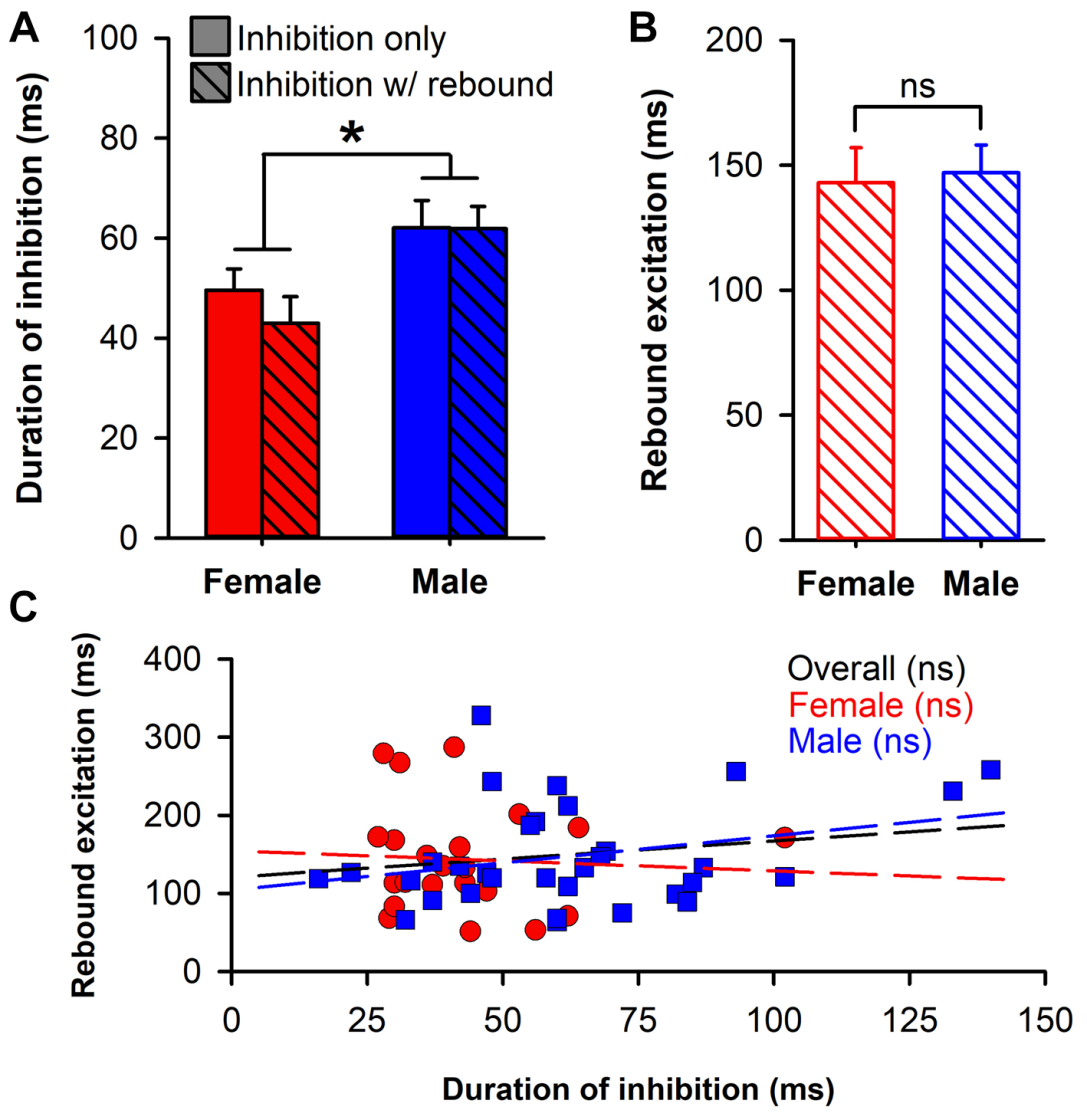
Analysis of rebound excitation. **(A)** Bar chart comparing duration of inhibition in two response phenotypes (inhibition only, solid; inhibition with rebound excitation, hatched) by sex (female, red; male, blue). Only the main effect of sex was significant (*, *p* = 0.002). **(B)** Bar chart comparing duration of rebound excitation in neurons from female (red) and male (blue) rats, which was not significantly different. **(C)** Scatterplot of duration of inhibition against duration of rebound excitation for neurons from female (red circles) and male (blue squares) rats. Overlaid lines represent regression lines for female (red) and male (blue) neurons, as well as the overall regression (black), none of which were significant.

To further explore the higher prevalence of rebound excitation in neurons from male rats, we compared the initial duration of inhibition in the two response phenotypes (inhibition only and inhibition with rebound) by sex (Figure 3A). There was a significant main effect of sex [*F*_(1,110)_ = 10.24, *p* = 0.002] with longer durations of inhibition in male rats. However, neither the main effect of response phenotype [F_(1,110)_ = 0.49, *p* = 0.488] nor the sex by phenotype interaction [F_(1,110)_ = 0.415, *p* = 0.521] were significant. Next, we compared the duration of rebound excitation by sex (Figure 3B) but found no significant difference [t_(54)_ = 0.21, *p* = 0.838]. Lastly, we tested whether an association between each phase of the response (inhibition and rebound excitation) was present using Pearson’s r (Figure 3C). The correlations between duration of inhibition and rebound excitation were not significant overall [r_(54)_ = 0.18, *p* = 0.179], amongst neurons from female rats (*r*_(21)_ = −0.06, *p* = 0.770), nor amongst neurons from male rats [r_(31)_ = 0.30, *p* = 0.090]. Although neither was individually significant, we lastly compared the correlation coefficients between neurons from female and male rats using a Fisher’s *z*-transformation and found no significant difference between the two (*z* = −1.29, *p* = 0.196). This suggests that while rebound excitation in neurons from male rats was more prevalent, this outcome was independent from the intensity of the initial inhibition.

### 3.2. Neuronal density in the RMTg is similar between female and male rats

**Figure 4:**
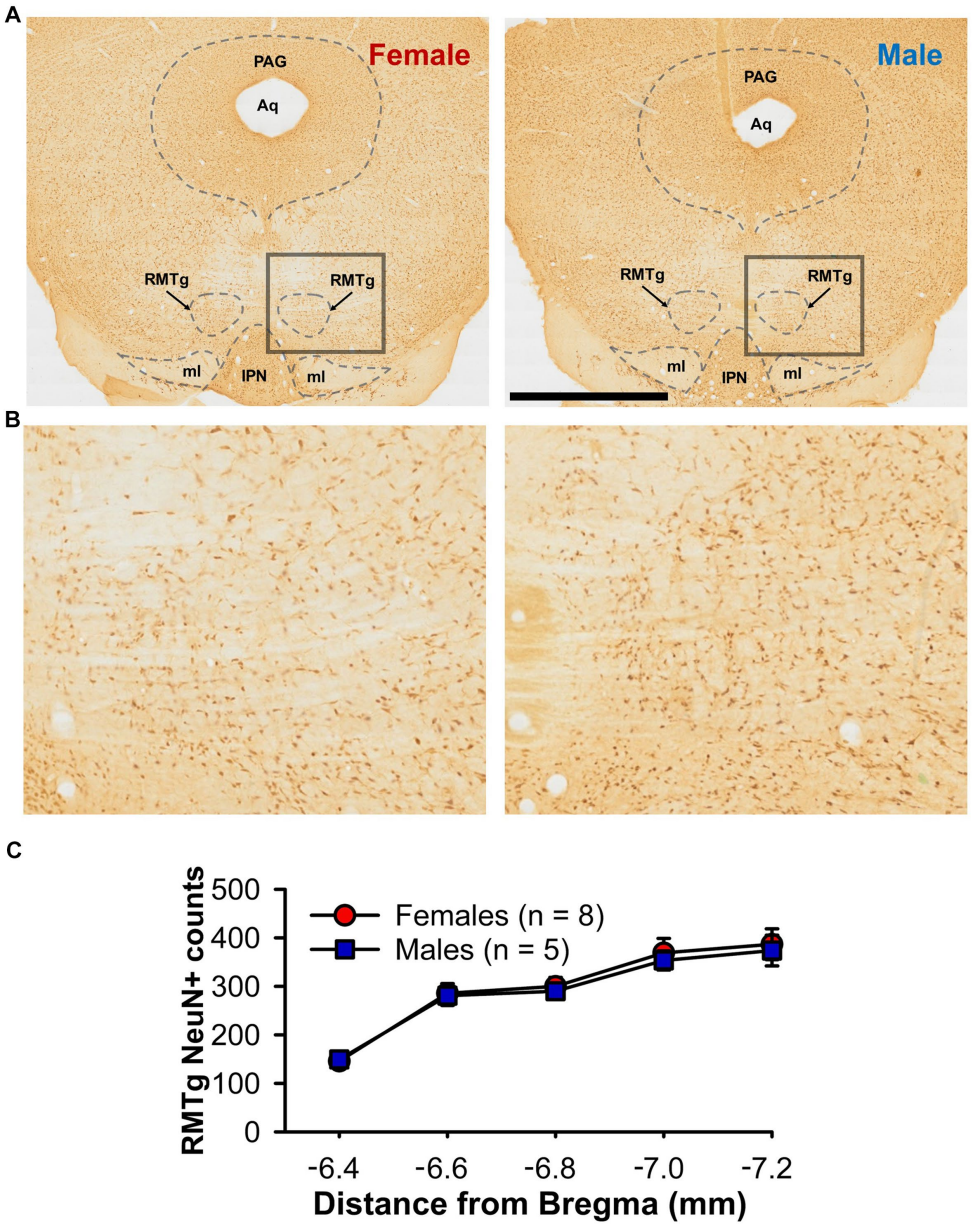
NeuN+ counts within the RMTg **(A)** Representative photomicrographs of immunochemistry for NeuN in the RMTg of a female (left) and male (right) rat at approximately bregma −6.7 mm **(B)** Higher magnification view of the boxed area from A. **(C)** Line graph showing bilateral NeuN+ counts through the rostral-caudal extent of the RMTg for both female (red) and male (blue) rats. Aq = cerebral aqueduct, IPN, interpeduncular nucleus; ml, medial lemniscus, PAG, periaqueductal gray; RMTg, rostromedial tegmental nucleus. Scale bar = 2 mm **(A)**, 0.5 mm **(B)**.

As LHb-induced inhibition of DA neurons is largely dependent upon feed-forward inhibition from the RMTg (Brown et al., 2017), we conducted counts of RMTg cells that positively immunostained for the neuron specific protein NeuN on a subset of female and male rats, testing the hypothesis that there is reduced neuronal density in female relative to male rats (Figures 4A,B). Overall neuron counts increased in the rostral-caudal direction [*F*_(4,44)_ = 58.33, *p* < 0.001; Figure 4C] as previously shown (Brown et al., 2017). However, there were no sex [*F*_(1,11)_ = 0.08, *p* = 0.788] nor sex by rostral-caudal section interaction effects [F_(4,44)_ = 0.10, *p* = 0.982], suggesting that neuronal density of the RMTg does not account for the sex-difference in LHb-induced inhibition of DA neurons.

### 3.3. Baseline LHb neuronal firing is more variable in male than female rats

**Figure 5:**
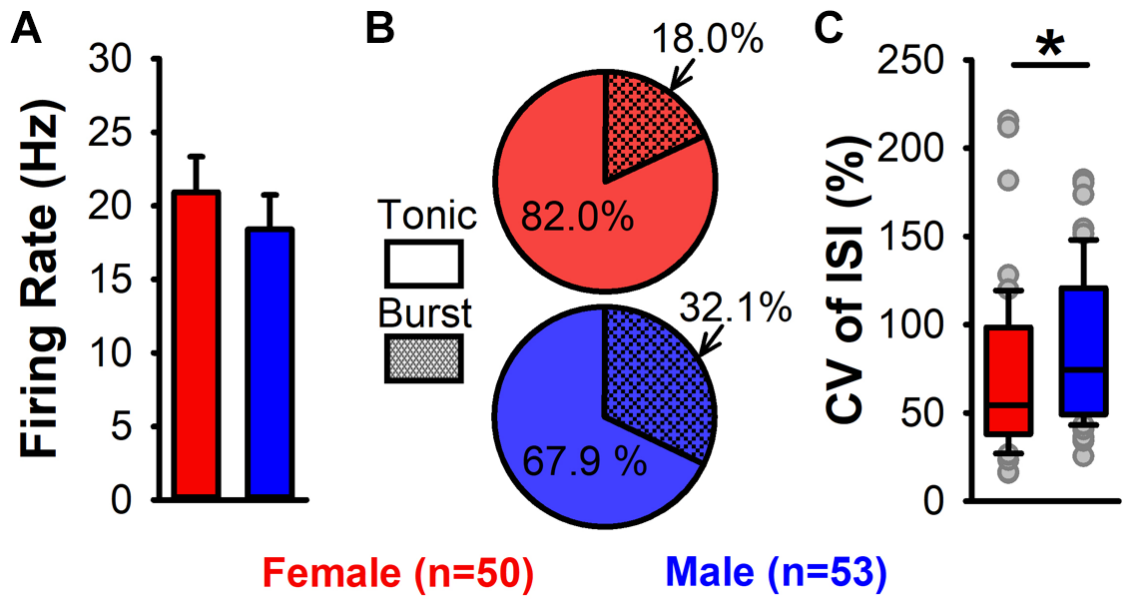
LHb neuron firing activity in female and male rats. **(A)** Mean firing rate of LHb neurons in female (red) and male (blue) rats. No significant difference in firing rate by sex was found. **(B)** Pie charts of tonic (solid) and burst (hatched) firing LHb neurons from female (red) and male (blue) rats. No significant difference in firing pattern by sex was found. **(C)** Box plots showing CV of ISI for female (red) and male (blue) rats. Female rats had a significantly lower CV of ISI (*, *p* = 0.02), demonstrating greater LHb firing regularity.

One hypothesis that may explain the sex difference in LHb-induced inhibition of DA neuron firing is that LHb neurons differ by sex in their excitability and therefore in their response duration or intensity to electrical stimulation. To test this, we conducted anesthetized recordings of a total of 103 LHb neurons (50 from female and 53 from male rats). Neurons from female and male rats did not differ in mean LHb firing rate [*t*_(101)_ = 0.7, *p* = 0.46; Figure 5A]. To assess firing variability of LHb neurons, we used two approaches: firing pattern analysis and CV of ISI. Though LHb neurons from female rats did show a lower prevalence of burst firing, this did not reach significance [*Χ*^2^ = 2.01, *p* = 0.16; Figure 5B]. However, the CV of ISI of LHb neurons was significantly lower in LHb neurons from female compared to male rats (median = 54.2% vs. 73.4%, Mann–Whitney *U* = 971, *p* = 0.02; Figure 5C), demonstrating that LHb neurons from male rats have more variability in firing.

### 3.4. Density of ERα + cells in the LHb is elevated in female rats

**Figure 6:**
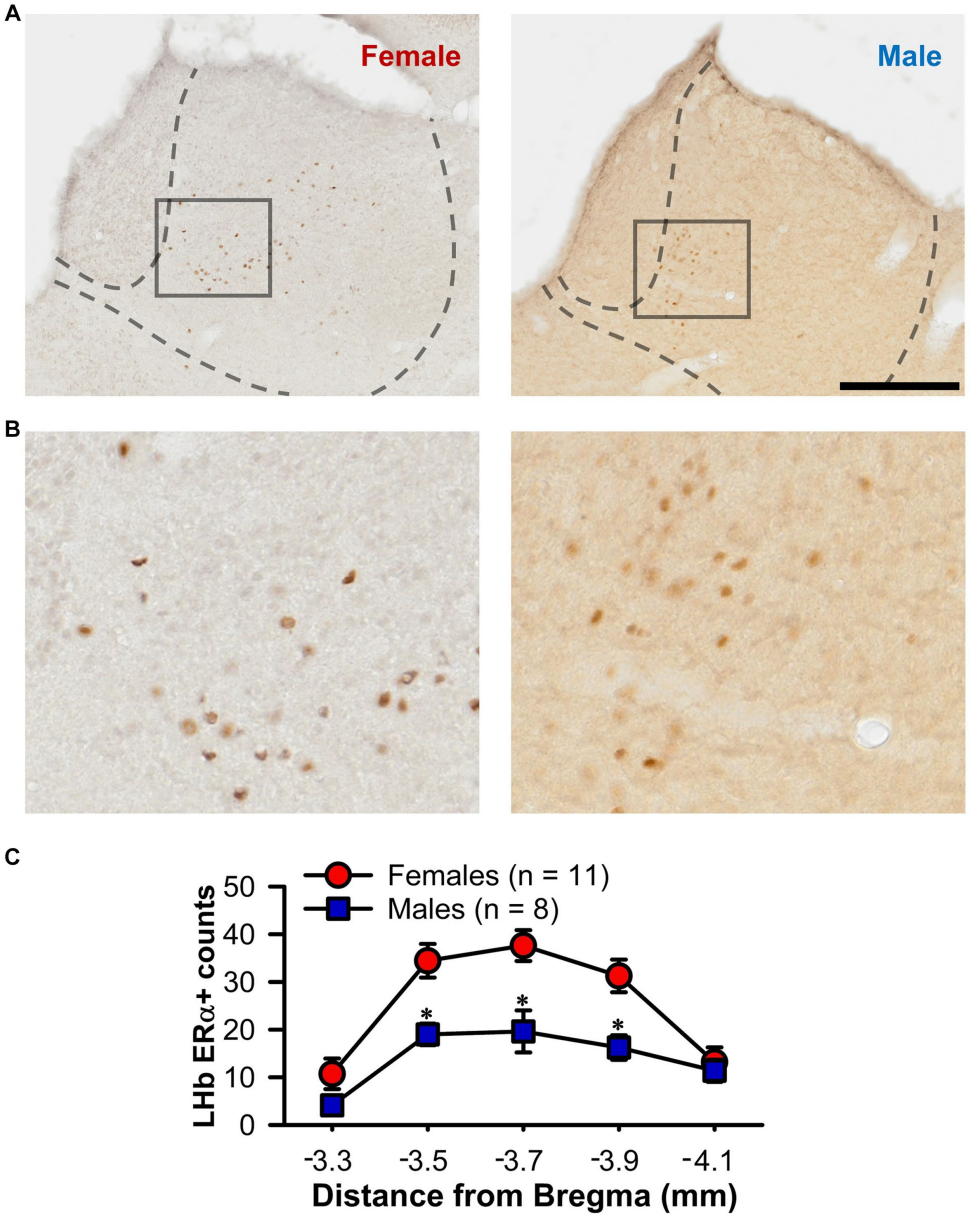
ERα + counts within the LHb. **(A)** Representative photomicrographs of immunochemistry for ERα in the LHb of a female (left) and male (right) rat. **(B)** Higher magnification view of the boxed area from A. **(C)** Line graph showing unilateral ERα + counts through the rostral-caudal extent of the LHb for both female (red) and male (blue) rats. Female rats showed a higher count of ERα + cells overall and the specific sections marked (*, *p* < 0.05). Scale bar = 500 μm **(A)**, 125 μm **(B)**.

The presence of a sex difference in LHb firing regularity raises the possibility that circulating gonadal hormones may exert an influence. To further explore this possibility, we conducted counts of ERα + cells through the rostral-caudal extent of the LHb in female and male rats (Figures 6A,B). A two-way ANOVA determined an effect of section number [*F*_(4,68)_ = 18.89, *p* < 0.001; Figure 6C], with the central portion of the LHb showing the highest number of positive cells (at −3.5, −3.7, and − 3.9 mm from bregma; Tukey test, *p* < 0.05). Furthermore, not only did female rats have a higher number of ERα + LHb cells overall [*F*_(1,17)_ = 22.33, *p* < 0.001], there was also a significant sex by section number interaction [*F*_(4,68)_ = 2.53, *p* = 0.048] with post-hoc tests demonstrating significant sex differences in the three central sections (Tukey test, *p* < 0.05).

### 3.5. Sex differences remain when accounting for stimulus and recording locations, and with the exclusion of medial VTA neurons

**Figure 7:**
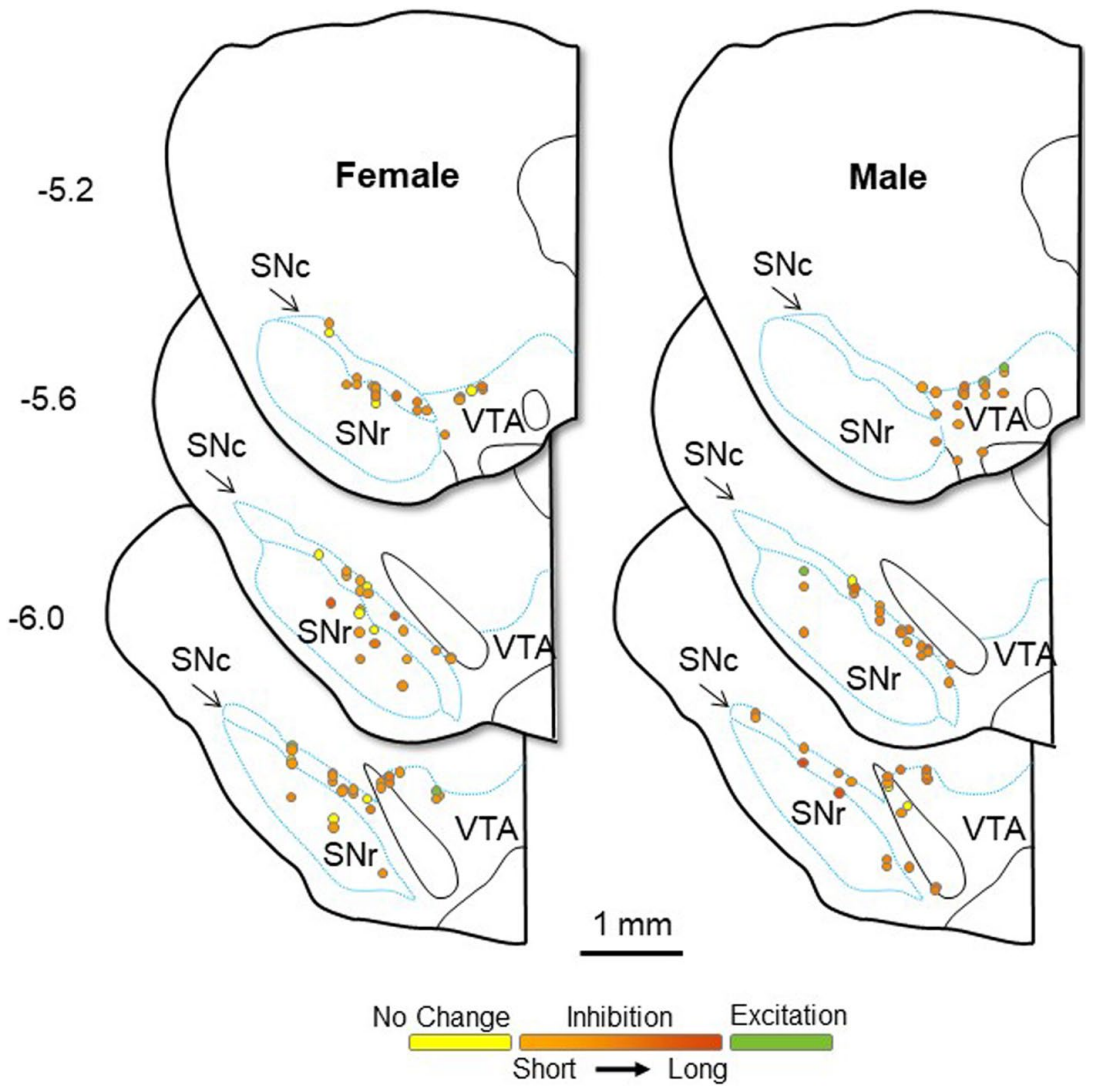
Approximate location of all DA neurons recorded in female (left) and male (right) rats, calculated from the location of dye spots and superimposed on coronal drawings at −5.2, −5.6, and − 6.0 mm from bregma. All neurons were electrophysiologically identified as DA neurons within the bounds of the SN or lateral VTA. Neurons are color coded by their response to LHb stimulation: excitation (green), no change (yellow), or inhibition (orange). The duration of inhibition is represented by the intensity of orange saturation. SNc, substantia nigra pars compacta; SNr, substantia nigra pars reticulata; VTA, ventral tegmental area.

**Figure 8:**
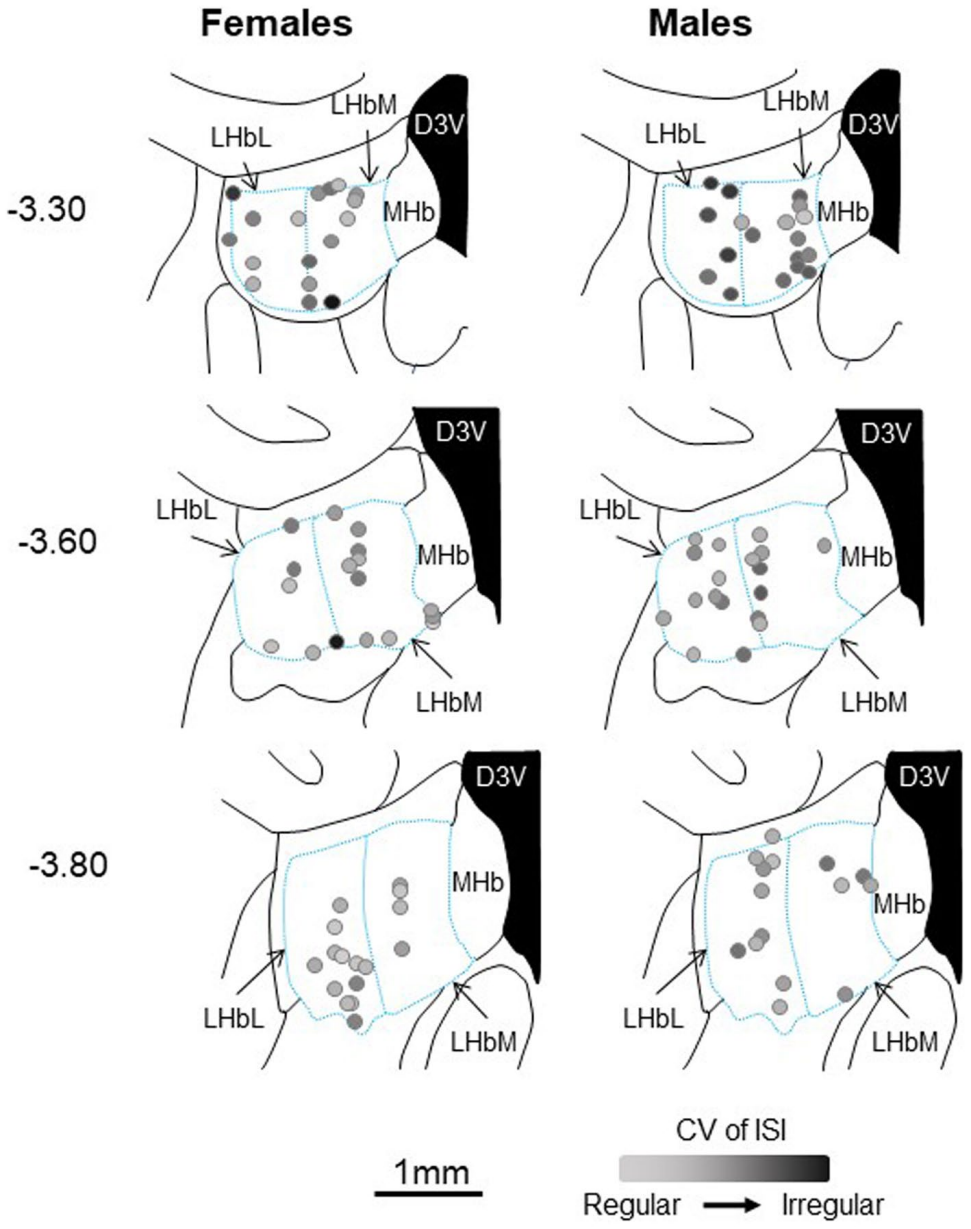
Approximate location of all LHb neurons recorded in female (left) and male (right) rats, calculated from the location of dye spots and superimposed on coronal drawings at −3.3, −3.6, and − 3.8 mm from bregma. All neurons were within the bounds of the LHb. For each neuron, the CV of ISI is represented by the intensity of black saturation. D3V, dorsal third ventricle; LHbL, lateral habenula, lateral portion; LHbM, lateral habenula, medial portion; MHb, medial habenula.

Given the possibility that the sex differences seen here could result from group differences in recording location, we performed multiple linear regressions to test if four predictor variables (the calculated three-axis location for each recorded neuron and sex) significantly predicted our response variables. Using this four-predictor model (RC-, ML-, and DV-coordinates; Sex) the overall regression for duration of inhibition in DA neurons was significant [*F*_(4,109)_ = 3.56, *p* = 0.009; *R*^2^ = 0.12; Figure 7]. The RC-coordinate was a significant predictor (*t* = 2.07, *p* = 0.041) with caudal neurons showing greater inhibition, as was sex (*t* = 2.97, *p* = 0.004) with males showing greater inhibition. Neither the ML- (*t* = 0.39, *p* = 0.70) nor the DV-coordinate (*t* = 1.01, *p* = 0.315) were significant predictors of duration of inhibition. The overall regression for the Box-Cox transformed CV of ISI in LHb neurons was significant (*F*_(4,98)_ = 9.13, *p* < 0.001; *R*^2^ = 0.27; Figure 8). Significant predictors were the RC-coordinate (*t* = 5.33, *p* < 0.001), the DV-coordinate (*t* = 2.14, *p* = 0.035), and sex (*t* = 2.31, *p* = 0.023), with rostral neurons, ventral neurons, and neurons from males showing greater CV of ISI. The ML-coordinate was not a significant predictor of CV of ISI (*t* = 0.47, *p* = 0.639). We used the same approach to test whether the location of the stimulating electrode may account for variability in the duration of inhibition of DA neurons. The overall regression in this case was significant [F_(4,109)_ = 3.61, *p* = 0.008; *R*^2^ = 0.12]. Sex was the sole significant predictor (*t* = 3.00, *p* = 0.003) with males showing greater inhibition; none of the axis coordinates were significant predictors (RC, *t* = 0.87, *p* = 0.385; ML, *t* = 0.49, *p* = 0.624; DV, *t* = 0.97, *p* = 0.336). Therefore, even when accounting for stimulus and recording location, male and female rats differ in baseline and evoked firing properties of the LHb-RMTg-DA pathway.

Finally, there is concern that electrophysiological identification of DA neurons in the VTA may inadvertently include non-DA neurons in any analysis, especially within the most medial portions (Ungless and Grace, 2012). To minimize this possibility, we reanalyzed the duration of inhibition excluding DA neurons located 1.0 mm or less from the midline. With this more stringent criterion, which excluded five and nine neurons from female and male rats respectively, the sex difference remained [*t*_(98)_ = 3.041, *p* = 0.003] with DA neurons from male rats showing a longer duration of inhibition.

## 4. Discussion

### 4.1. Main findings

The experiments presented here provide several novel findings. First, while the well-demonstrated ability of the LHb to inhibit DA neuronal firing is confirmed, this inhibition is relatively weaker in female rats (quantitively different), and lacks the rebound excitation commonly seen in male rats (qualitatively different). This finding is in line with previous research that showed a sex difference in RMTg-induced inhibition of VTA neurons (Melis et al., 2013). Since LHb inhibition of DA neuron firing runs largely through the RMTg (Brown et al., 2017) and RMTg activation is largely dependent upon LHb input (Brown and Shepard, 2013; Jhou et al., 2013) our study likely reflects the same inhibitory phenomenon. The difference does not appear to be due to sex differences in RMTg neuronal density (Figure 4), but this conclusion is tempered by the fact that the GABAergic nature of these neurons was not confirmed. Although strain differences may play a role, the weaker inhibition seen by others may be due to the rather disbursed nature of the RMTg (Jhou et al., 2009) making activation of the RMTg by electrical stimulation as a whole, either directly on cell bodies or indirectly through LHb axon terminals, difficult. In addition, the prevalence of rebound excitation in male rats was found to be greater. LHb-induced inhibition is similar between neurons with and without rebound excitation and is unaffected by sex (Figure 3A). Furthermore, the duration of rebound excitation is similar between female and male rats (Figure 3B), and there is no significant correlation between initial inhibition and rebound excitation (Figure 3C). It appears that the lower prevalence of rebound excitation in female rats is not a result of reduced initial inhibition but is simply due to there being fewer cells with this type of response phenotype. Collectively, these data provide evidence of a significant sex difference in the inhibitory strength of the LHb-RMTg-DA pathway.

Second, while there were no differences in LHb neuronal firing rate, the firing of LHb neurons was more regular in female rats. This is in keeping with previous work demonstrating that estrogen downregulates the expression and permeability of Cav3.3 calcium channels in the LHb (Song et al., 2018), which contribute to hyperpolarization-induced burst firing in LHb neurons (Weiss and Veh, 2011). A higher propensity for LHb burst firing following stimulation would explain the greater inhibition in DA firing seen here in males. In addition, LHb burst firing is generally followed by a refractory period (Weiss and Veh, 2011), which may partially explain the greater rebound excitation of DA neurons in male rats since, during such a refractory period, the LHb role as an active inhibitory “brake” on DA firing would have been temporarily removed (Barrot et al., 2012). Although the present study did not manipulate concentrations of circulating estrogen, the overall higher levels of estrogen in female rats (Overpeck et al., 1978) would lead one to predict the difference in firing regularity seen. One limitation of the present study is that estrous stage was not established prior to recording. This added variability may have reduced the intensity of the sex differences seen here. Accounting for this factor may point toward the potential influence of circulating gonadal hormones on this neurocircuit.

Third, ERα + cells are more prevalent in the LHb of female rats than male rats. In addition to the commonly known ERα and ERβ, which exist in both cytosolic and membrane-bound forms (Arnal et al., 2017), there are several other membrane associated ERs (Toran-Allerand, 2005; Barton et al., 2018). We looked specifically for sex differences in the distribution of ERα + LHb cells in adult rats because previous work on this receptor was limited to either single sex studies (Shughrue et al., 1997; Lonstein et al., 2000) or the early post-natal developmental period (Yokosuka et al., 1997; Pérez et al., 2003). More importantly, however, ERα + LHb neurons with local axon collaterals were recently shown to project to the midbrain (Zhang et al., 2018a) and are activated by maternal behavior (Lonstein et al., 2000). Although the role of these neurons is unclear, the potential exists that estrogen acts here to alter the inhibitory influence of the LHb on midbrain monoaminergic centers and consequently behavioral outcomes such as reward seeking and stress responsivity (Zhang et al., 2018a).

### 4.2. Limitations

There are caveats associated with the electrophysiological identification of DA neurons. However, this is primarily a concern when sampling from the medial / rostral VTA, a concern that does not extend to the SN (Ungless and Grace, 2012). Furthermore, immunochemically identified neurons from the lateral VTA overwhelmingly have the same properties used to electrophysiologically identify DA neurons including a slower firing rate, wider action potential and longer time constant when compared to immunochemically identified non-DA neurons (Brown and Shepard, 2016). Given that we sampled almost exclusively from the SN and lateral VTA and used stringent guidelines for electrophysiological identification, and that the sex difference remained when medial VTA neurons were excluded from the analysis, the possibility of misidentification of DA neurons has been minimized.

In the statistical design of this experiment, we have focused on the neuron as the behavioral “unit of interest.” This raises the possibility that, when sampling multiple neurons from individual animals, underlying subject characteristics (i.e., estrous phase, developmental history) affect the independence of these outcomes. To minimize this, we have followed recommendations for the design of such electrophysiological experiments including recording from a sufficiently large sample of animals; recording relatively consistent numbers of neurons from each animal; and reporting the number of animals, neurons, and neurons/animal (Recommendations for the design …, 2018).

Lastly, in an anesthetized preparation the behavior of DA neurons may be altered by the anesthetic used and the depth of anesthesia, with different advantages and disadvantages for each compound. Chloral hydrate maintains burst firing, but this fidelity is susceptible to the depth of anesthesia; isoflurane is easily administered but may elevate firing and bursting; ketamine, due to its antagonism of NMDA receptors, is not appropriate if studying burst firing (Marinelli and McCutcheon, 2014). The compound used here, urethane, has the advantage that it maintains a very stable plane of anesthesia, but reduces firing rate (Marinelli and McCutcheon, 2014) and makes DA neuronal firing susceptible to changes in brain-state (Walczak and Błasiak, 2017). Therefore, there is the possibility that these results may not extend to other anesthetized preps or the awake animal.

### 4.3. Conclusion

These data add to a growing body of evidence that the LHb, although not sexually dimorphic (McCarthy et al., 2012), displays significant sex differences. Anatomically, the LHb receives a larger innervation of glutamatergic input from hypothalamic areas in female mice but a larger GABAergic input from the medial septum in males (Liu et al., 2022b). Stress induces sex-dependent activation of the LHb (Sood et al., 2018; Kim and Chung, 2021) and of the direct LHb-VTA pathway (Zhang et al., 2018b), and sex-dependent stress sensitivity appears to be driven by activation of ERα containing hypothalamic projections to the LHb (Calvigioni et al., 2023). Behaviorally in mice, pup retrieval in females requires activation of LHb neurons, which show sexual differentiation in parental-behavior associated gene expression (Lecca et al., 2023) and social communication is regulated in a sex-dependent manner by LHb vasopressin receptors (Rigney et al., 2020). Furthermore, the direct action of estrogen also points toward a significant sex-difference in the LHb-RMTg-DA pathway. In addition to the downregulation of LHb Cav3.3 calcium channel expression (Song et al., 2018), estrogen exposure reduces LHb neuronal activation (Li et al., 2015), and local administration of an ERβ agonist, diarylpropionitrile, to the LHb reverses ovariectomy-induced increases in both behavioral measures of anxiety and baseline LHb cFos production (Liu et al., 2022a). Further work is needed to show whether these sex differences, as well as the ones described presently, result from hormone-dependent activational effects, organizational effects of development, or some other undetermined process. Determining those causative factors will have broad applications to a number of neurological or mental health disorders in which sex is considered a contributing factor.

## Data availability statement

The raw data supporting the conclusions of this article will be made available by the authors, without undue reservation.

## Ethics statement

The animal study was approved by the University of Maryland Baltimore IACUC. The study was conducted in accordance with the local legislation and institutional requirements.

## Author contributions

DB: Data curation, Investigation, Methodology, Writing – review & editing, Formal analysis, Visualization. VW: Data curation, Investigation, Methodology, Visualization, Writing – review & editing. PB: Conceptualization, Data curation, Formal analysis, Funding acquisition, Investigation, Methodology, Project administration, Resources, Supervision, Writing – original draft, Writing – review & editing, Visualization.
